# Inhibition of G-Protein βγ Signaling Decreases Levels of Messenger RNAs Encoding Proinflammatory Cytokines in T Cell Receptor-Stimulated CD4^+^ T Helper Cells

**DOI:** 10.5334/1750-2187-10-1

**Published:** 2015-07-06

**Authors:** Thomas R. Hynes, Evan A. Yost, Cassandra M. Hartle, Braden J. Ott, Catherine H. Berlot

**Affiliations:** Weis Center for Research, Geisinger Clinic, Danville, Pennsylvania, 17822-2623, United States of America

**Keywords:** heterotrimeric G-protein βγ complex, T helper cells, Cytokine, STAT4, IFN-γ, IL-17A

## Abstract

**Background:** Inhibition of G-protein βγ (Gβγ) signaling was found previously to enhance T cell receptor (TCR)-stimulated increases in interleukin 2 (IL-2) mRNA in CD4^+^ T helper cells, suggesting that Gβγ might be a useful drug target for treating autoimmune diseases, as low dose IL-2 therapy can suppress autoimmune responses. Because IL-2 may counteract autoimmunity in part by shifting CD4^+^ T helper cells away from the Type 1 T helper cell (TH1) and TH17 subtypes towards the TH2 subtype, the purpose of this study was to determine if blocking Gβγ signaling affected the balance of TH1, TH17, and TH2 cytokine mRNAs produced by CD4^+^ T helper cells.

**Methods:** Gallein, a small molecule inhibitor of Gβγ, and siRNA-mediated silencing of the G-protein β_1_ subunit (Gβ_1_) were used to test the effect of blocking Gβγ on mRNA levels of cytokines in primary human TCR-stimulated CD4^+^ T helper cells.

**Results:** Gallein and Gβ_1_ siRNA decreased interferon-γ (IFN-γ) and IL-17A mRNA levels in TCR-stimulated CD4^+^ T cells grown under TH1-promoting conditions. Inhibiting Gβγ also decreased mRNA levels of STAT4, which plays a positive role in TH1 differentiation and IL-17A production. Moreover, mRNA levels of the STAT4-regulated TH1-associated proteins, IL-18 receptor β chain (IL-18Rβ), mitogen-activated protein kinase kinase kinase 8 (MAP3K8), lymphocyte activation gene 3 (LAG-3), natural killer cell group 7 sequence (NKG7), and oncostatin M (OSM) were also decreased upon Gβγ inhibition. Gallein also increased IL-4, IL-5, IL-9, and IL-13 mRNA levels in TCR-stimulated memory CD4^+^ T cells grown in TH2-promoting conditions.

**Conclusions:** Inhibiting Gβγ to produce these shifts in cytokine mRNA production might be beneficial for patients with autoimmune diseases such as rheumatoid arthritis (RA), Crohn’s disease (CD), psoriasis, multiple sclerosis (MS), and Hashimoto’s thyroiditis (HT), in which both IFN-γ and IL-17A are elevated.

## Introduction

We demonstrated previously that blocking Gβγ signaling resulted in potentiation of TCR-stimulated IL-2 mRNA increases in human CD4^+^ T helper cells [1], suggesting this approach might be useful for treating autoimmune diseases, as low dose IL-2 therapy effectively suppressed immune responses in chronic graft-versus-host disease [2] and hepatitis C virus-induced vasculitis [3]. Because IL-2 regulates the differentiation of the TH1, TH2, and TH17 CD4^+^ T helper cell subsets [4,5,6], we hypothesized that part of the mechanism by which IL-2 counteracts autoimmunity could be via an alteration in the balance of these cell types and the cytokines that they produce. TH1, TH2, and TH17 CD4^+^ T helper cell subsets mediate unique immunological functions, but defects in their functions can also contribute to immune disorders. TH1 cells protect against intracellular organisms, but can also cause inflammation and autoimmune diseases, whereas TH2 cells protect mucosal and epithelial surfaces, but can also cause allergy and asthma [7]. Recently recognized TH17 cells [8] are required for mucosal immunity, but can also produce autoimmunity by producing proinflammatory cytokines [9]. Previous studies support a role for IL-2 in shifting CD4^+^ T Helper cells away from the TH1 and TH17 subtypes towards the TH2 subtype. For instance, IL-2 complexes ameliorated autoantibody-mediated autoimmunity in an experimental model for myasthenia gravis both by expanding regulatory T cells and by causing a switch from TH1 to TH2 responses [10,11]. Moreover, IL-2/STAT5 signaling leads to decreased TH17 responses [12,13].

Antagonizing or deleting multiple G-protein-coupled receptors (GPCRs) can induce a shift away from the TH1 and TH17 T helper cell subsets and counteract autoimmunity. For instance, antagonizing the EP_4_ receptor for prostaglandin E_2_ inhibited TH1 differentiation and TH17 expansion and was orally active in arthritis models [14], and experimental autoimmune encephalomyelitis (EAE) and contact hypersensitivity [15]. Deletion of the histamine receptor type 1 (H1R) in mice resulted in decreased production of IFN-γ, increased production of IL-4, and protection from EAE and experimental autoimmune orchitis (EAO) [16]. Mice lacking the CC chemokine receptor CCR2 exhibited decreased antigen-stimulated IFN-γ production and were resistant to EAE [17]. Deficiency of the TH1-associated CC chemokine receptor CCR5 [18], resulting from a deletion variant of the CCR5 gene (CCR5Δ32), protected against inflammation-associated mortality in dialysis patients [19] and decreased the risk of developing acute graft-versus-host disease after allogenic hematopoietic stem cell transplantation [20]. Antagonism of the TH1-associated chemokine, IP-10, which binds the CXC chemokine receptor CXCR3, decreased IFN-γ production and ameliorated the progression of autoimmune sialadenitis in MRL/*lpr* mice [21]. Blocking the signaling of these GPCRs could have applications for TH1/TH17 shifted diseases, but as multiple GPCRs are involved in promoting the TH1 and TH17 subsets, targeting signaling distal to these GPCRs, such as at the level of heterotrimeric G-proteins, could also be advantageous.

Downstream of GPCRs, G protein α subunits have been implicated in modulating the balance of CD4^+^ T helper cell subsets. For instance, selective deletion of Gα_s_ from CD4^+^ T cells resulted in impaired differentiation of TH1 and TH17 cells, whereas TH2 and regulatory T cells were unaffected [22]. T cells isolated from Gα_q_-deficient mice had altered TCR responses, including reduced LAT phosphorylation, sustained ERK1/2 phosphorylation, and increased secretion of IL-2, IL-5, IL-12, and TNF-α [23]. Mice lacking Gα_i2_ developed a TH1-mediated inflammatory colitis [24] and their CD4^+^ T cells exhibited enhanced responses to TCR signaling [25] and were defective in chemokine receptor signaling, chemotaxis, and homing [26].

The purpose of this study was to determine if blocking Gβγ signaling affects the balance of cytokine mRNA levels in primary human TCR-stimulated CD4^+^ T helper cells. We determined previously that targeting Gβγ with a small molecule inhibitor, gallein, and siRNA directed at Gβ_1_ enhanced TCR-stimulated IL-2 transcription [1] in these cells. Gallein is a member of a class of Gβγ inhibitors, of which M119 is the prototype, that specifically blocks interactions between Gβγ, but not Gα, with effectors, and does not promote dissociation of Gα from Gβγ [27]. Although relatively little is known about the role of Gβγ complexes in modulating T cell signaling, gallein/M119 has been used successfully in animal models to inhibit neutrophil chemotaxis and inflammation [28], to potentiate morphine-induced analgesia [27], and to inhibit the progression of heart failure [29]. These precedents suggested that targeting Gβγ might provide an effective way to block signaling from the multiple GPCRs that can promote TH1 and/or TH17 differentiation. Indeed, this study demonstrates that inhibiting Gβγ in TCR-stimulated CD4^+^ T helper cells decreases levels of mRNAs encoding IFN-γ and IL-17A, while increasing levels of TH2 cytokine mRNAs.

## Methods

### Ethics statement and study population

This study was reviewed and approved by the Geisinger Health System Internal Review Board, and all study participants signed informed consent. Peripheral blood was obtained from 30 healthy women 18 to 70 years old who did not have any autoimmune, infectious, or atopic diseases, clinical suspicion of anemia, or treatment with greater than 10 mg of prednisone within 12 hours of the blood draw. The peripheral blood samples used in this study were the same as those used in our previous study [1].

### Isolation and culture of human CD4^+^ T cells

Peripheral blood mononuclear cells (PBMCs) were isolated using Ficoll-Paque density gradient centrifugation. CD4^+^ T cells were isolated by depletion of non-CD4^+^ T cells using a CD4^+^ T Cell Isolation Kit II (Miltenyi Biotec). The cells were then separated into naïve and memory CD4^+^ T cells using a Naïve CD4^+^ T cell Isolation Kit (Miltenyi Biotec). Purification of the cells was confirmed by labeling samples before and after purification with fluorescently labeled antibodies to either CD4 and CD45RA (to label naïve cells) or CD4 and CD45RO (to label memory cells) and analysis using flow cytometry. 94.3% of the cells in the naïve T cell preparations were CD4^+^ (SE = 0.7%, ranging from 83.9% to 98.6%) and 83.8% were CD45RA^+^ (SE = 1.4%, ranging from 68.1% to 95.9%). 95.2% of the cells in the memory T cell preparations were CD4^+^ (SE = 0.4%, ranging from 89.7% to 98%) and 75.0% were CD45RO^+^ (SE = 1.8%, ranging from 55.0% to 88.6%). Cells were plated in 24-well dishes coated with 2.5 µg/ml anti-CD3 antibody (Miltenyi) in RPMI containing 10% FCS, 2.5 µg/ml anti-CD28 antibody (Miltenyi) and IL-2 (2 ng/ml) (R&D Systems). For TH1 differentiation, the media also included 20 ng/ml IL-12 and 1 µg/ml anti-IL-4 antibody (R&D Systems). For TH2 differentiation, the media also included 20 ng/ml IL-4 and 2 µg/ml anti-IL-12 antibody (R&D Systems). Cells were harvested after three days.

### siRNA and gallein treatments

siRNAs were produced by Dharmacon. The sequence of Gβ_1_ siRNA (GGAUAACAUUUGCUCCAUU) is from [30]. The non-targeting (NT) siRNA used was ON-TARGETplus Non-targeting Pool (Dharmacon, D-001810-10-20).

siRNAs were introduced into primary CD4^+^ T cells by nucleofection using a Nucleofector II Device (amaxa/Lonza). 2–9 × 10^6^ primary CD4^+^ T cells were nucleofected with 10 µM siRNA using 100 µL of Human T Cell Nucleofector Solution and Program U-014. After nucleofection, the primary CD4^+^ T cells were incubated in RPMI with 10% FCS for 6 hours before transfer to activating/differentiating media and further incubation for three days.

Gallein and fluorescein (TCI America) were used at a final concentration of 15 µM and were added when the cells were placed in activating/differentiating media.

### Quantitative PCR (qPCR)

RNA was prepared using RNeasy Plus Mini Kits (Qiagen). cDNA was prepared using QuantiTect Reverse Transcription kits (Qiagen). QPCR was performed using TaqMan Gene Expression Assays (Applied Biosystems) and an Applied Biosystems qPCR machine. mRNA expression levels were determined by comparing the C_t_ value of the mRNA of interest to that of the house-keeping gene GAPDH in the same preparation.

### Statistics

The significance of effects of siRNAs, gallein, and fluorescein on primary CD4^+^ T cells was determined using the Wilcoxon signed rank test (paired, non-parametric). Values of *p* < 0.05 were considered significant (^*^, *p* < 0.05; ^**^, *p* < 0.01; ^***^, *p* < 0.001; ^****^, *p* < 0.0001).

## Results

### Gallein, a small molecule inhibitor of Gβγ signaling, and siRNA-mediated silencing of Gβ_1_ decrease IFN-γ mRNA levels in human TCR-stimulated CD4^+^ T cells grown under TH1-promoting conditions

We determined previously that blocking Gβγ signaling in primary human CD4^+^ T helper cells grown in TH1- or TH2-promoting conditions enhanced TCR-stimulated IL-2 mRNA levels by ~2-fold [1]. As low dose IL-2 therapy has shown promise for treating autoimmune diseases, effectively suppressing immune responses in chronic graft-versus-host disease [2] and hepatitis C virus-induced vasculitis [3], and part of the mechanism by which IL-2 counteracts autoimmunity may involve shifting CD4^+^ T helper cells from the TH1 and TH17 subtypes to the TH2 subtype [10,11,12,13,31,32], we sought to determine whether blocking Gβγ signaling affected the balance between these T helper cell subtypes. To this end, we investigated whether gallein, a small molecule inhibitor of Gβγ signaling [28], and siRNA directed at Gβ_1_ affected the relative expression of mRNAs encoding TH1, TH2, and TH17 cytokines in CD4^+^ T helper cells. We focused on cytokine mRNAs because transcriptional regulation is the primary means of controlling expression of inducible cytokine genes [33,34,35,36,37,38] and because our own measurements of qPCR-determined IL-2 mRNA levels and secreted IL-2 [1] and those of others [39] demonstrated a strong correlation, and similar relationships have been determined for IFN-γ [40], IL-17 [41], and TH2 cytokines [42,43].

**Figure 1:**
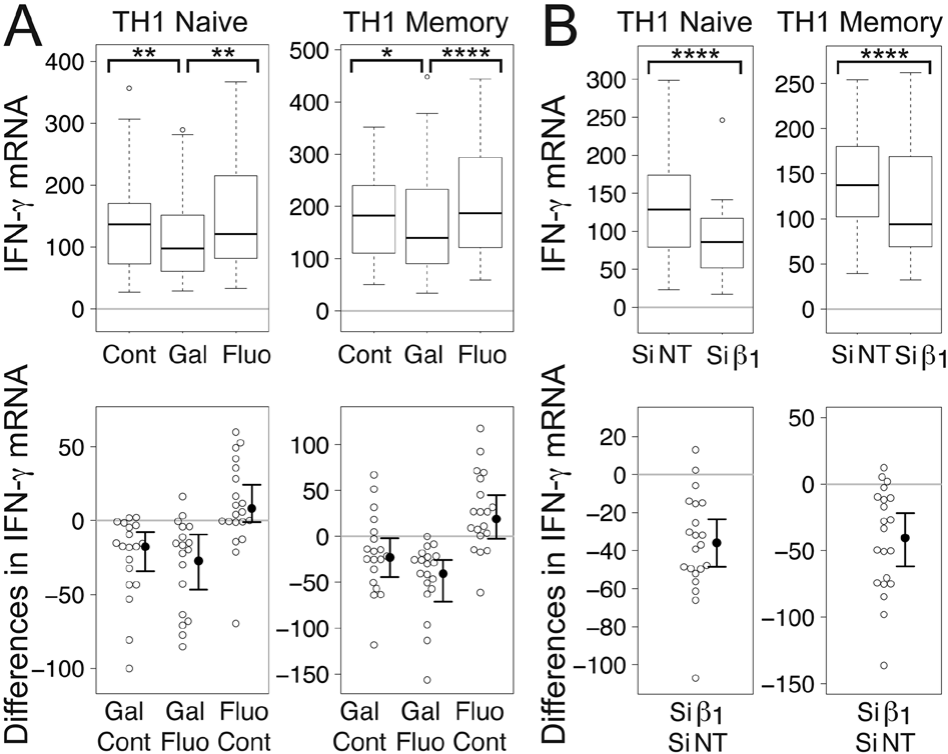
**Gβγ inhibition decreases IFN-γ mRNA levels in TCR-stimulated CD4^+^ TH1 cells.** (A) Gallein, but not fluorescein, significantly decreased IFN-γ mRNA levels. Box plots (top) and difference plots (bottom) show data from naïve (left) and memory (right) CD4^+^ T cells isolated from the peripheral blood of 20 healthy donors, stimulated with plate-bound anti-CD3 and soluble anti-CD28, and grown in conditions promoting TH1 differentiation in the absence or presence of gallein or fluorescein for three days. In the box plots (top), the height of the box plots equals the interquartile range (IQR) and the horizontal line within the box indicates the median value. The whiskers extend to the lowest and highest data points within 1.5 X IQR and the open circles indicate the outliers. In the difference plots (bottom), open circles show pairwise differences in IFN-γ mRNA for each sample when treated with the top versus bottom condition listed on the X axis. To the right of the open circles are the median values (closed circles) and 95% confidence intervals. (B) Gβ_1_ siRNA significantly decreased IFN-γ mRNA levels. Box plots (top) and difference plots (bottom) show data from naïve (left) and memory (right) CD4^+^ T cells isolated from the blood of 30 healthy donors and stimulated for three days with plate-bound anti-CD3 and soluble anti-CD28 in conditions promoting TH1 differentiation in the presence of Gβ_1_ siRNA or NT siRNA. IFN-γ mRNA levels were determined by qPCR. ^*^, *p* < 0.05; ^**^, *p* < 0.01; ^****^, *p* < 0.0001.

We focused first on IFN-γ, one of the prototypical TH1 cytokines, which plays an important role in TH1 differentiation [44], in naïve and memory CD4^+^ T cells grown under TH1-promoting conditions and stimulated at the TCR by plate-bound anti-CD3 antibodies and soluble anti-CD28 antibodies. Gallein significantly decreased mean levels of IFN-γ mRNA in both naïve and memory TH1 cells to levels that were 87% and 71%, respectively, of control levels (Fig. 1A). In contrast, fluorescein, a structurally related but inactive compound [28], did not have a significant effect (Fig. 1A). M119, a Gβγ inhibitor that is structurally and functionally similar to gallein [28], and which operates by the same mechanism [45], blocks the interactions of Gβγ with downstream effectors, but does not interfere with GPCR-dependent Gα activation or Gα-effector interactions [27]. Our results therefore indicate that Gβγ plays a role in increasing IFN-γ mRNA levels in TCR-stimulated TH1 cells that is downstream or independent of GPCR-Gα signaling.

Similar to the effect of gallein, Gβ_1_ siRNA significantly decreased mean levels of IFN-γ mRNA in naïve and memory TCR-stimulated TH1 cells to levels that were 71% and 74%, respectively, of levels in the presence of NT siRNA (Fig. 1B). We showed previously that Gβ_1_ and Gβ_2_ accounted for >99% of the total Gβ subunit mRNAs in primary human naïve and memory CD4^+^ T cells and that a second Gβ_1_ siRNA had similar effects on Gβ_1_ and TCR-stimulated IL-2 mRNAs as the one used here [1]. These results indicate that Gβ_1_γ complexes play a role in increasing IFN-γ mRNA levels in TCR-stimulated TH1 cells.

The magnitude of these effects of Gβγ inhibition on IFN-γ mRNA levels is similar to that of variations in IFN-γ production that are relevant to autoimmune diseases. For instance, the number of IFN-γ-secreting CD4^+^ T cells was ~1.25-fold higher in patients with secondary progressive MS compared to controls [46]. Moreover, treatment of MS patients with antibodies to IFN-γ for 5 consecutive days, which lowered IFN-γ levels to 70% of the starting level after one month and 85% of the starting level after 6 months, caused a significant decrease in the number of patients with disease progression during a 9–12 month period after treatment, as well as a significant increase in the time without progression, compared to the placebo group [47]. Additionally, the mean concentration of IFN-γ produced by CD4^+^ T cell clones from gut tissue biopsy specimens from CD patients was 1.23-fold higher than that produced by clones from patients with noninflammatory gut disorders [48]. Furthermore, one study found that patients with HT exhibited a 1.47-fold increase in the number of CD4^+^ IFN-γ^+^ PBMCs [49] and another reported that HT patients had 1.49-fold higher serum levels of IFN-γ [50].

### Gallein and Gβ_1_ siRNA decrease IL-17A mRNA levels in human TCR-stimulated CD4^+^ memory T cells grown under TH1-promoting conditions

There is precedent for the existence of TH17/TH1 cells that produce both IL-17 and IFN-γ [51,52,53,54]. Accordingly, although we did not grow CD4^+^ T cells in TH17-promoting conditions, we investigated whether TCR-stimulated CD4^+^ T cells grown in TH1-promoting conditions produced IL-17A, and if so, whether levels were affected by Gβγ inhibition, because Gβγ inhibition enhanced TCR-stimulated IL-2 transcription in CD4^+^ T cells grown in TH1-promoting conditions [1], and IL-2 inhibits IL-17 production [12,13]. Of the two highly homologous IL-17 family members, IL-17A and IL17F, we focused on IL-17A, because, unlike IL-17F, IL-17A appears to mediate autoimmunity [9,55]. Additionally, we focused on memory cells, because IL-17 is produced primarily if not exclusively in activated CD4^+^ memory rather than naïve T cells [56].

**Figure 2:**
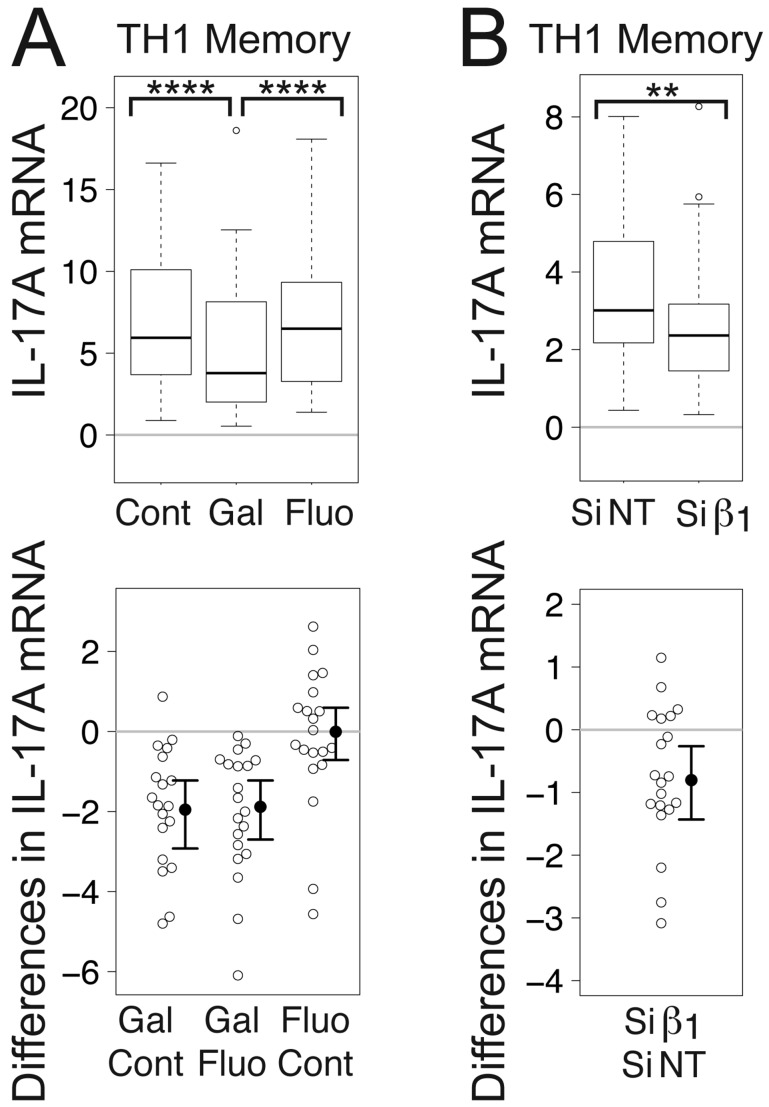
**Gβγ inhibition decreases IL-17A mRNA levels in TCR-stimulated CD4^+^ TH1 memory cells.** (A) Gallein, but not fluorescein, significantly decreased IL-17A mRNA levels. Box plots (top) and difference plots (bottom) show data from CD4^+^ memory T cells isolated from the peripheral blood of 20 healthy donors, stimulated with plate-bound anti-CD3 and soluble anti-CD28, and grown in conditions promoting TH1 differentiation in the absence or presence of gallein or fluorescein for three days. (B) Gβ_1_ siRNA significantly decreased IL-17A mRNA levels. Box plots (top) and difference plots (bottom) show data from memory CD4^+^ T cells isolated from the blood of 30 healthy donors and stimulated for three days with plate-bound anti-CD3 and soluble anti-CD28 in conditions promoting TH1 differentiation in the presence of Gβ_1_ siRNA or NT siRNA. IL-17A mRNA levels were determined by qPCR. ^**^, *p* < 0.01; ^****^, *p* < 0.0001.

Gallein, but not fluorescein, significantly decreased the mean level of IL-17A mRNA in TCR-stimulated TH1 memory cells to 71% of the control value (Fig. 2A). Similarly, Gβ_1_ siRNA significantly decreased the mean level of IL-17A mRNA to 54% of the level in the presence of NT siRNA (Fig. 2B). To put these results in perspective, one study found that serum levels of IL-17A were 2.28-fold higher in RA patients compared to osteoarthritis (OA) patients [57]. Another study comparing TH17 cells from RA patients versus healthy controls demonstrated a 3-fold increase in the amount of IL-17A produced by the cells from the RA patients [58]. Additionally, the number of lymphocytes expressing IL-17 from patients with relapsing relapse-remitting MS (RRMS) was approximately 3-fold higher than in controls and remitting RRMS [54]. Consistent with this, another study found that the number of PBMCs expressing IL-17 mRNA was 2.8-fold higher in patients with MS compared to healthy controls [59].

### Gallein and Gβ_1_ siRNA decrease levels of STAT4 mRNA in human TCR-stimulated CD4^+^ T cells grown under TH1-promoting conditions

We observed previously that potentiation of IL-2 transcription required continuous Gβγ inhibition during at least two days of TCR stimulation and that this potentiation was obtained only after IL-2 levels had decreased from an initial peak [1]. These results could indicate that Gβγ signaling plays a role in the negative feedback mechanisms that result in the transient nature of IL-2 secretion in TCR-stimulated CD4^+^ T cells [60,61,62,63]. As T helper cells differentiate into TH1 cells, the IL-12 that they produce inhibits IL-2 production by a mechanism involving the transcription factor STAT4 [60,64]. In addition, IL-18 synergizes with IL-12 to induce expression of IFN-γ in T cells [65] and enhance TH1 differentiation [66,67], and IL-12-mediated induction of an IL-18 receptor (IL-18R) complex and the IL-18Rβ subunit requires STAT4 [68]. Moreover, STAT4 plays a role in the development of IL-23-primed IL-17-secreting cells, and is required for IL-17 production in response to IL-23 plus IL-18 [69]. Mice lacking STAT4 have lower levels of IL-17 than control animals [70].

**Figure 3:**
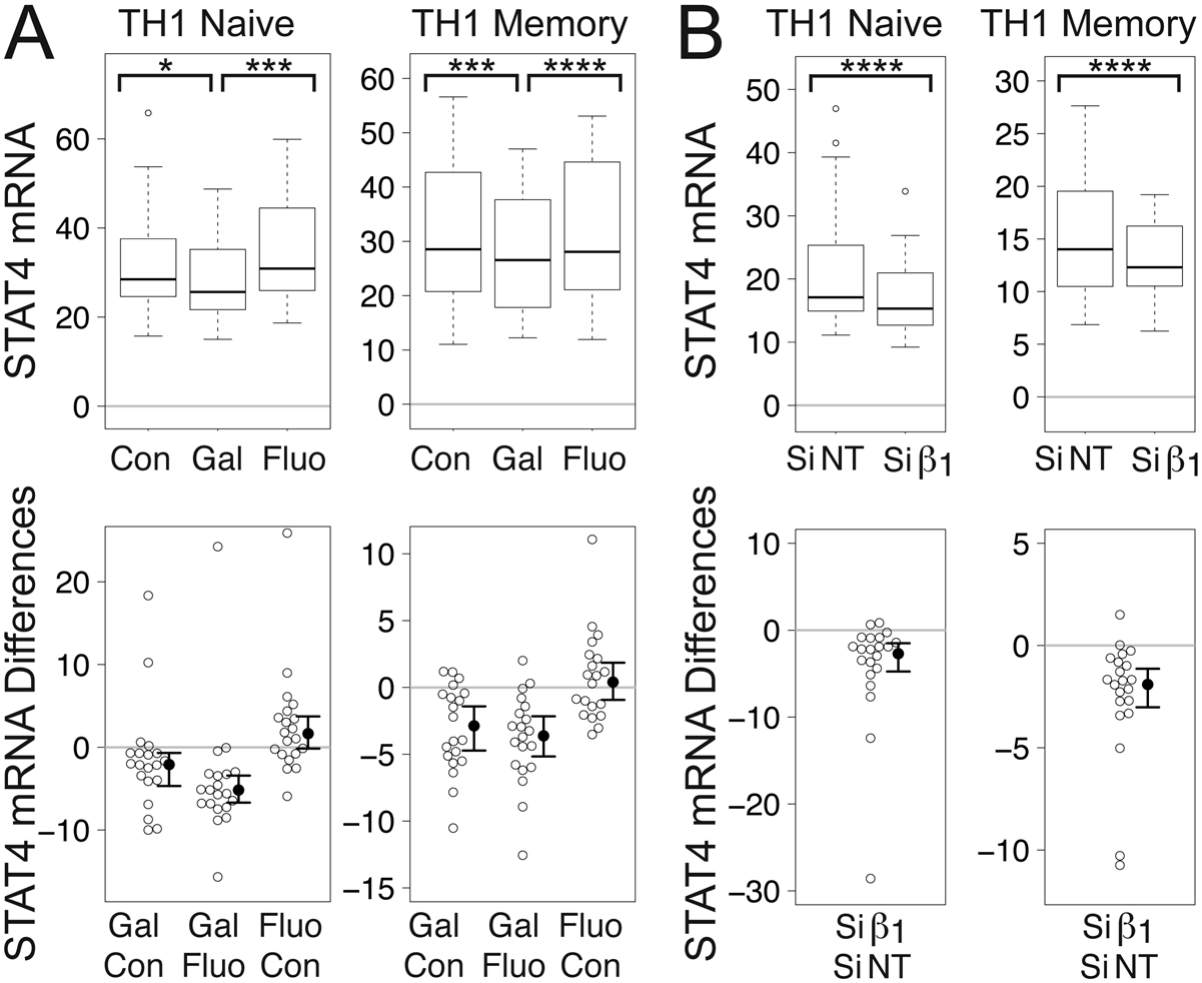
**Gβγ inhibition decreases STAT4 mRNA in TCR-stimulated CD4^+^ TH1 cells.** (A) Gallein, but not fluorescein, significantly decreased STAT4 mRNA levels. Box plots (top) and difference plots (bottom) show data from naïve (left) and memory (right) CD4^+^ T cells isolated from the peripheral blood of 20 healthy donors, stimulated with plate-bound anti-CD3 and soluble anti-CD28, and grown in conditions promoting TH1 differentiation in the absence or presence of gallein or fluorescein for three days. (B) Gβ_1_ siRNA significantly decreased STAT4 mRNA levels. Box plots (top) and difference plots (bottom) show data from primary human naïve (left) and memory (right) CD4^+^ T cells isolated from the blood of 30 healthy donors and stimulated for three days with plate-bound anti-CD3 and soluble anti-CD28 in conditions promoting TH1 differentiation in the presence of Gβ_1_ siRNA or NT siRNA. STAT4 mRNA levels were determined by qPCR. ^*^, *p* < 0.05; ^***^, *p* < 0.001; ^****^, *p* < 0.0001.

For the above reasons, we investigated whether levels of STAT4 mRNA were decreased upon inhibition of Gβγ signaling. Indeed, gallein, but not fluorescein, significantly decreased mean levels of STAT4 mRNA in both naïve and memory TCR-stimulated TH1 cells to levels that were 91% and 93%, respectively, of control levels (Fig. 3A). Similarly, Gβ_1_ siRNA significantly decreased mean levels of STAT4 mRNA in TCR-stimulated naïve and memory TH1 cells to levels that were 81% and 84%, respectively, of those in the presence of NT siRNA (Fig. 3B).

The decreases in IFN-γ mRNA (Fig. 1) and IL-17A mRNA (Fig. 2) in response to gallein and Gβ_1_ siRNA were generally larger than the decreases in STAT4 mRNA (Fig. 3). Therefore, while decreased STAT4 activity resulting from Gβγ inhibition is likely to contribute to the decreases in these cytokine mRNAs, this decreased STAT4 activity probably does not account entirely for the decreased cytokine mRNAs. Additional changes in response to Gβγ inhibition, such as enhanced levels of TCR-stimulated IL-2 [1] (see Discussion), are likely to be involved as well.

### Gallein and Gβ_1_ siRNA decrease mRNA levels of STAT4-regulated genes in human TCR-stimulated CD4^+^T cells grown under TH1-promoting conditions

The modest decreases in STAT4 mRNA levels resulting from Gβγ inhibition were most likely too low to allow for detection of corresponding decreases in STAT4 protein levels. However, to determine if these STAT4 mRNA decreases might be physiologically significant and contribute to a shift away from the TH1 and TH17 T helper cell subsets, we investigated whether the expression of STAT4-regulated genes that contribute to these T helper cell subsets was correspondingly decreased. Several studies have identified STAT4 target genes that play a role in TH1 lineage-specific programming [71,72]. We investigated whether Gβγ inhibition in TCR-stimulated TH1 cells decreased mRNA levels of a subset of these genes, some of which also play roles in IL-17 production.

### Gallein and Gβ_1_ siRNA decrease levels of IL-18Rβ mRNA

STAT4 regulates expression of IFN-γ in part by regulating IL-12-mediated induction of an IL-18R complex that includes the IL-18Rβ subunit [68], which also plays a role in IL-17 production. Human PBMCs secrete pro-IL-18 [73], which is proposed to be cleaved extracellularly into mature IL-18 [74]. The role of IL-18 appears to be that of activating/amplifying IL-17 production in synergy with IL-23 in polarized TH17 cells [75]. For these reasons, we investigated the effect of Gβγ inhibition on IL-18Rβ mRNA expression.

**Figure 4:**
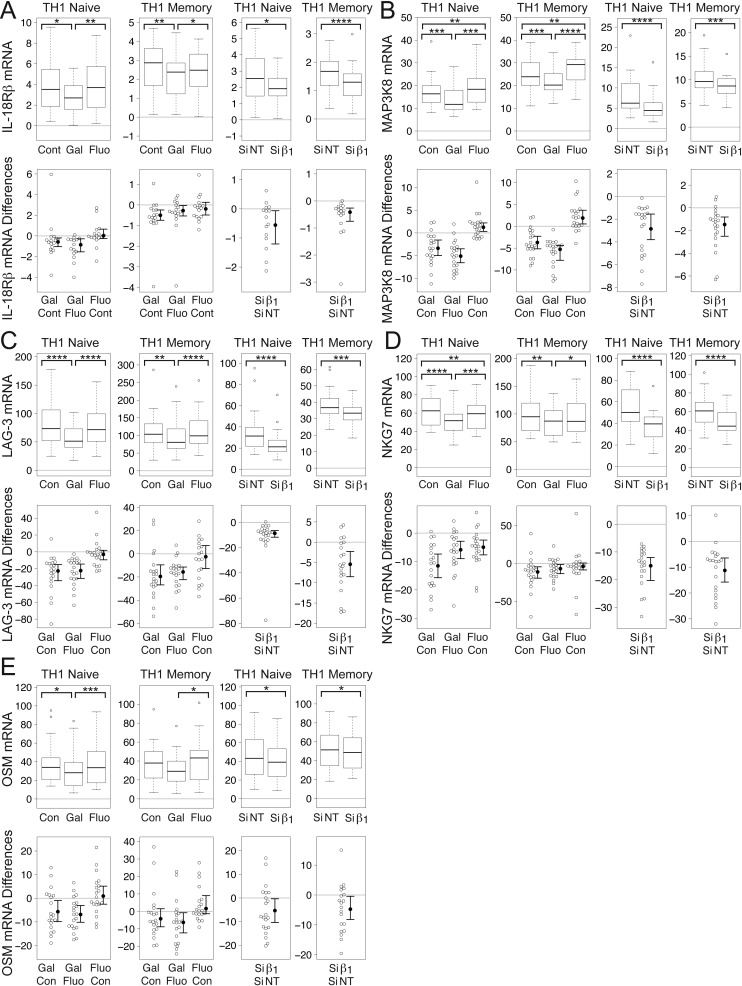
**Gβγ inhibition decreases levels of mRNA encoding STAT4-regulated genes in TCR-stimulated CD4^+^ TH1 cells.** Gallein, but not fluorescein, and Gβ_1_ siRNA significantly decreased mRNA levels of IL-18Rβ (A), MAP3K8 (B), and LAG-3 (C) in TCR-stimulated naïve and memory TH1 cells. (D) Gallein, but not fluorescein, significantly decreased NKG7 mRNA levels in TCR-stimulated memory TH1 cells. Gallein significantly decreased NKG7 mRNA levels in naïve cells, but fluorescein also caused a significant, but smaller decrease. Gβ_1_ siRNA significantly decreased NKG7 mRNA levels in both naïve and memory cells. (E) Gallein, but not fluorescein, significantly decreased OSM mRNA levels in TCR-stimulated naïve TH1 cells. Gallein decreased OSM mRNA levels significantly in memory cells relative to fluorescein-treated cells, but not to control cells. Gβ_1_ siRNA significantly decreased OSM mRNA levels in both naïve and memory cells. (A-E) First two sets of graphs: box plots (top) and difference plots (bottom) show data from naïve (left) and memory (right) CD4^+^ T cells isolated from the peripheral blood of 20 healthy donors, stimulated with plate-bound anti-CD3 and soluble anti-CD28, and grown in conditions promoting TH1 differentiation in the absence or presence of gallein or fluorescein for three days. Second two sets of graphs: box plots (top) and difference plots (bottom) show data from primary human naïve (left) and memory (right) CD4^+^ T cells isolated from the blood of 30 healthy donors and stimulated for three days with plate-bound anti-CD3 and soluble anti-CD28 in conditions promoting TH1 differentiation in the presence of Gβ_1_ siRNA or NT siRNA. mRNA levels were determined by qPCR. ^*^, *p* < 0.05; ^**^, *p* < 0.01; ^***^, *p* < 0.001; ^****^, *p* < 0.0001.

Gallein, but not fluorescein, significantly reduced mean levels of IL-18Rβ mRNA in naïve and memory TCR-stimulated TH1 cells to levels that were 89% and 78%, respectively, of control levels (Fig. 4A). Similarly, Gβ_1_ siRNA significantly decreased mean levels of IL-18Rβ mRNA in both naïve and memory TCR-stimulated TH1 cells to levels that were 67% of levels in the presence of NT siRNA (Fig. 4A). These decreases in IL-18Rβ mRNA correlate with and could contribute to the decreases in mRNA levels of both IFN-γ (Fig. 1) and IL-17A (Fig. 2) resulting from Gβγ inhibition.

### Gallein and Gβ_1_ siRNA decrease levels of MAP3K8 mRNA

MAP3K8, also known as Tumor progression locus 2 (Tpl2) and cancer Osaka thyroid oncogene (Cot), is induced in T cells by IL-12 in a STAT4-dependent manner, and deficiency is associated with impaired production of both IFN-γ and IL-17 [76]. Moreover, CD4^+^ T cells from mice lacking MAP3K8 exhibited diminished induction of STAT4 in response to TCR activation [76], indicative of a positive feedback loop between MAP3K8 and STAT4.

Gallein, but not fluorescein, significantly reduced mean levels of MAP3K8 mRNA in naïve and memory TCR-stimulated TH1 cells to levels that were 80% and 85%, respectively, of control levels (Fig. 4B). Fluorescein actually increased MAP3K8 mRNA in naïve and memory TH1 cells to levels that were 115% and 109%, respectively, of control levels. Similar to the effect of gallein, Gβ_1_ siRNA significantly decreased mean levels of MAP3K8 mRNA in naïve and memory TCR-stimulated TH1 cells to levels that were 66% and 83%, respectively, of levels in the presence of NT siRNA (Fig. 4B).

### Gallein and Gβ_1_ siRNA decrease levels of LAG-3 mRNA

*LAG-3* is a TH1 cell gene to which STAT4 binds, resulting in epigenetic modifications [71]. Surface LAG-3 expression correlated with IFN-γ production in antigen-stimulated T cells and was upregulated by IL-12 [77]. Release of soluble LAG-3-related peptides by activated CD4^+^ T cell clones correlated positively with IFN-γ production and inversely with IL-4 production [77].

Gallein, but not fluorescein, significantly reduced mean levels of LAG-3 mRNA in naïve and memory TCR-stimulated TH1 cells to levels that were 72% and 84%, respectively, of control levels (Fig. 4C). Similarly, Gβ_1_ siRNA significantly decreased mean levels of LAG-3 mRNA in naïve and memory TCR-stimulated TH1 cells to levels that were 68% and 86%, respectively, of levels in the presence of NT siRNA (Fig. 4C).

### Gallein and Gβ_1_ siRNA decrease levels of NKG7 mRNA

*NKG7*, which encodes a cell surface cytotoxic molecule also known as GMP-17 and TIA-1 [78,79], is bound and induced by STAT4, exhibits STAT4-dependent epigenetic modifications, and is preferentially expressed in TH1 cells [71]. Along with a number of other genes that comprise the “IFN-γ resistome”, *NKG7* is upregulated in mice infected with *Salmonella* [80]. It is thought to be critical for co-stimulatory signal transduction and lymphocyte activation [80], as well as regulation of target cells and termination of the immune response [81].

Gallein, but not fluorescein, significantly reduced mean levels of NKG7 mRNA in naïve and memory TCR-stimulated TH1 cells to levels that were 81% and 88%, respectively, of control levels (Fig. 4D). In naïve TH1 cells, fluorescein also significantly reduced mean levels of NKG7 mRNA, but the effect was smaller. Mean levels in the presence of fluorescein were 95% of the control levels. Similar to the effect of gallein, Gβ_1_ siRNA significantly decreased mean levels of NKG7 mRNA in naïve and memory TCR-stimulated TH1 cells to levels that were 65% and 81%, respectively, of levels in the presence of NT siRNA (Fig. 4D).

### Gallein and Gβ_1_ siRNA decrease levels of OSM mRNA

OSM is a proinflammatory cytokine expressed primarily in TH1 cells [82,83]. TH1 cells deficient in STAT4 secreted greatly reduced levels of OSM and exhibited decreased hematopoietic progenitor cell numbers and cycling status, and injection of OSM into STAT4-deficient mice restored progenitor cell activity to wild-type levels [82]. OSM synergized with IL-17 to induce collagen degradation in bovine cartilage explants [84] and in cartilage explants from human patients with RA [85]. Moreover, IL-17A and OSM synergistically induced skin inflammation that recapitulated some features of psoriasis [86].

Gallein, but not fluorescein, reduced mean levels of OSM mRNA in naïve and memory TCR-stimulated TH1 cells to levels that were 84% and 76%, respectively, of control levels (Fig. 4E). In naïve TH1 cells, the effect of gallein was significant compared to both fluorescein and the untreated control whereas in memory TH1 cells the effect was significant only when compared to fluorescein. Gβ_1_ siRNA significantly decreased mean levels of OSM mRNA in naïve and memory TCR-stimulated TH1 cells to levels that were 89% and 92%, respectively, of levels in the presence of NT siRNA (Fig. 4E).

## Gallein increases levels of IL-4, IL-5, IL-9, and IL-13 mRNA in human TCR-stimulated CD4^+^ memory T cells grown in TH2-promoting conditions

Given that Gβγ inhibition resulted in decreased levels of IFN-γ mRNA in TCR-stimulated CD4^+^ T cells grown under TH1 conditions (Fig. 1) and that TH1 and TH2 cytokines mutually inhibit each other’s production [87], we investigated whether Gβγ inhibition also affected TH2 cytokines in TCR-stimulated CD4^+^ T cells grown under TH2 conditions. We measured IL-4, a TH2 cytokine that also induces TH2 differentiation [88,89], IL-5 and IL-13, which are produced primarily by activated TH2 cells [34,90], and IL-9, long thought to be a TH2 cytokine, because it promotes allergic inflammation and is associated with TH2 responses [91,92], but which is now also known to be produced by TH9, TH17, T regulatory (T_Reg_), mast, and natural killer cells [93].

**Figure 5:**
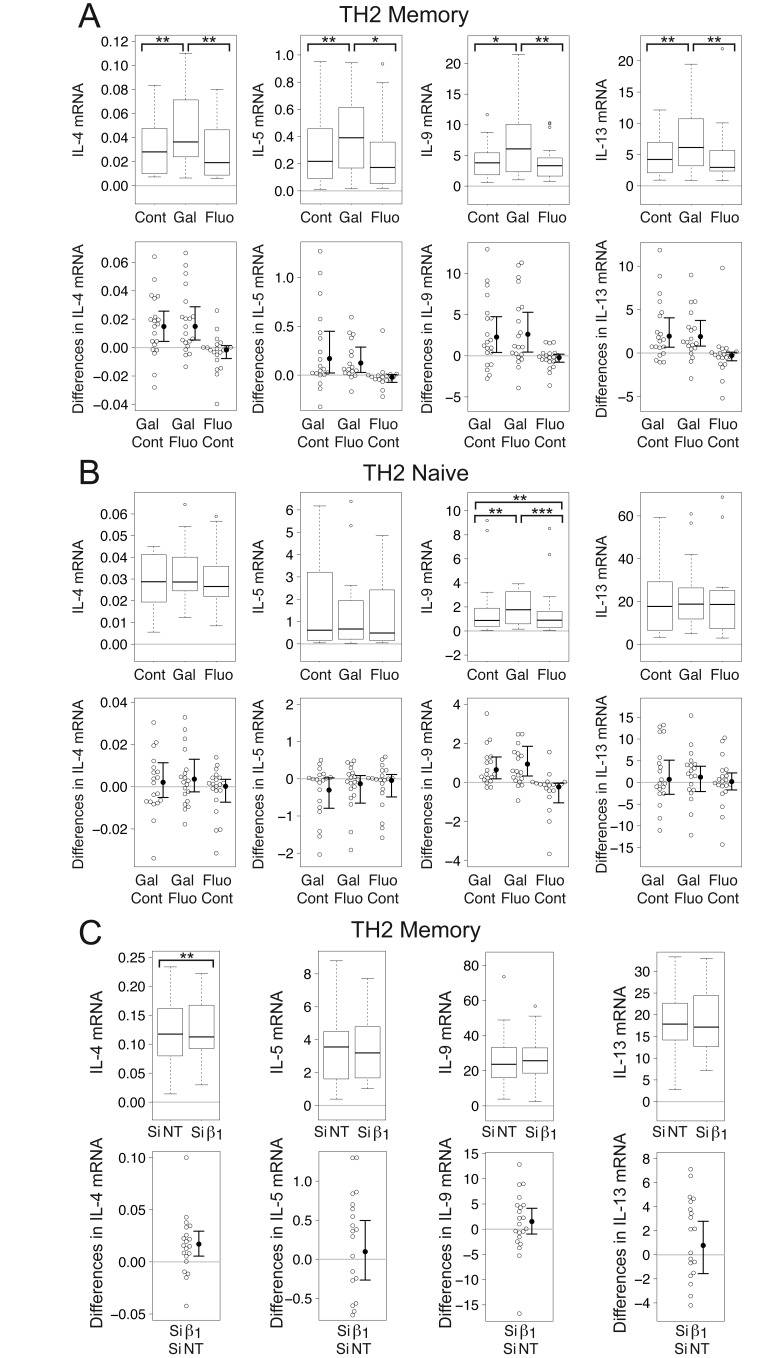
**Gβγ inhibition increases levels of mRNA encoding TH2 cytokines in TCR-stimulated CD4^+^ TH2 memory cells.** (A) Gallein, but not fluorescein, significantly increased levels of IL-4, IL-5, IL-9, and IL-13 mRNA in TCR-stimulated TH2 memory cells. (B) Gallein, but not fluorescein, significantly increased IL-9, but not IL-4, IL-5, or IL-13 mRNA levels in TCR-stimulated TH2 naive cells. (C) Gβ_1_ siRNA significantly increased IL-4, but not IL-5, IL-9, or IL-13 mRNA levels in TCR-stimulated TH2 memory cells. (A-B) Box plots (top) and difference plots (bottom) show data from memory (A) and naive (B) CD4^+^ T cells isolated from the peripheral blood of 20 healthy donors, stimulated with plate-bound anti-CD3 and soluble anti-CD28, and grown in conditions promoting TH2 differentiation in the absence or presence of gallein or fluorescein for three days. (C) Box plots (top) and difference plots (bottom) show data from memory CD4^+^ T cells isolated from the blood of 30 healthy donors and stimulated for three days with plate-bound anti-CD3 and soluble anti-CD28 in conditions promoting TH2 differentiation in the presence of Gβ_1_ siRNA or NT siRNA. mRNA levels were determined by qPCR. ^*^, *p* < 0.05; ^**^, *p* < 0.01; ^***^, *p* < 0.001.

Gallein significantly enhanced mean levels of mRNA encoding IL-4 (1.46-fold), IL-5 (1.62-fold), IL-9 (1.63-fold), and IL-13 (1.53-fold) in TCR-stimulated TH2 memory cells relative to control stimulated cells (Fig. 5A), but only significantly enhanced mean levels of IL-9 mRNA (1.32-fold) in TCR-stimulated TH2 naïve cells (Fig. 5B). To put these results in perspective, serum IL-4 levels in psoriatic patients were 84% of those in healthy controls [94]. Moreover, the median level of IL-13 in first-degree relatives of insulin-dependent diabetes mellitus (IDDM) subjects who were at high risk of IDDM was 87% of that of a control group whereas that in subjects with lower genetic risk was 1.24-fold higher [95].

As the predominant effect of gallein on TH2 cytokines was in memory cells, we investigated whether Gβ_1_ siRNA had a similar effect in these cells. Whereas Gβ_1_ siRNA significantly increased mean levels of IL-4 mRNA in TCR-stimulated TH2 memory cells by 1.18-fold, it did not affect levels of IL-5, IL-9, or IL-13 mRNA (Fig. 5C). As gallein is not known to discriminate among different Gβγ combinations, the greater magnitude of the effect of gallein compared to Gβ_1_ siRNA on IL-4 mRNA and the lack of effect of Gβ_1_ siRNA on levels of IL-5, IL-9, and IL-13 mRNA may indicate that Gβ subunit(s) other than or in addition to Gβ_1_ can decrease levels of these cytokines. The most likely possibility would be Gβ_2_, as Gβ_1_ and Gβ_2_ account for >99% of the total Gβ subunit mRNAs in primary naïve and memory CD4^+^ T cells [1].

## Gallein and Gβ_1_ siRNA decrease mRNA levels of STAT4 and several STAT4-regulated genes in TCR-stimulated CD4^+^ TH2 memory cells

Although STAT4 is activated in response to IL-12, and plays a pivotal role in differentiation of naïve CD4^+^ T cells to the TH1 subtype [96,97], its role in inhibiting production of IL-2 appears to be independent from IL-12. There is increased IL-2 production in STAT4^-/-^ CD4^+^ T cells even when IL-12 is not added (nonpolarizing TH0 condition)[64], and neutralizing IL-12 has little effect on IL-2 production [60]. Moreover, STAT4 antagonizes TH2 differentiation [97]. As Gβγ inhibition led to increases in levels of TH2 cytokine mRNAs in TCR-stimulated TH2 memory cells, we investigated whether Gβγ inhibition also decreased STAT4 mRNA levels in these cells.

**Figure 6:**
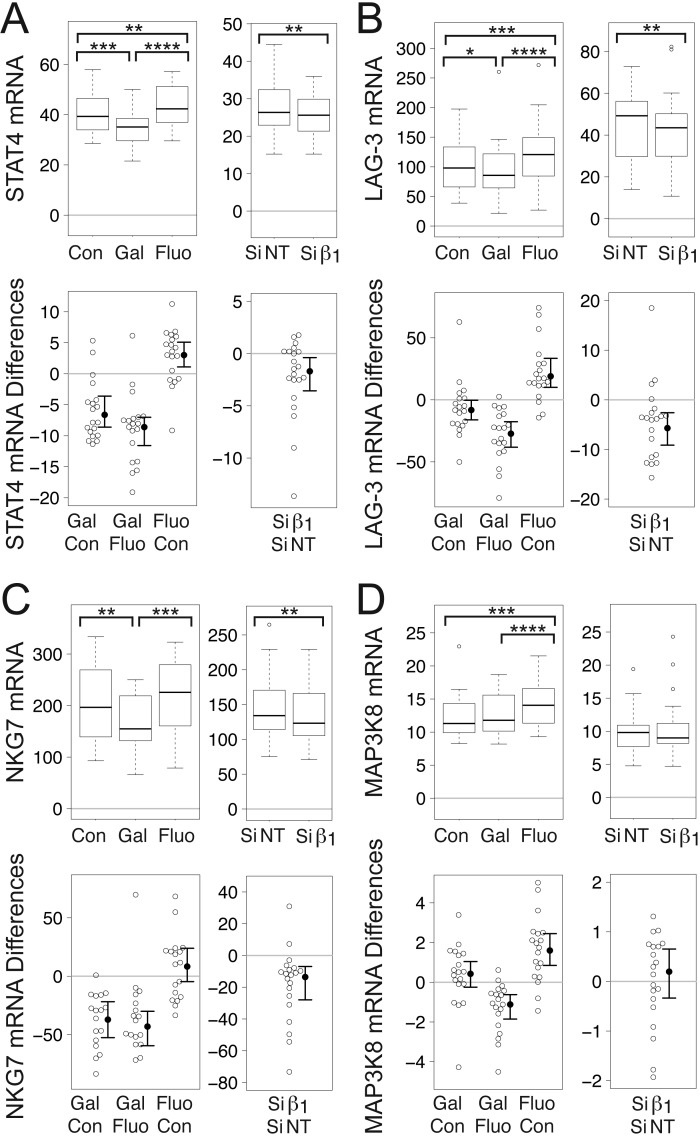
**Gβγ inhibition decreases mRNAs encoding STAT4 and some STAT4-regulated genes in CD4^+^ TH2 memory cells.** (A) Gallein, but not fluorescein, and Gβ_1_ siRNA significantly decreased levels of mRNA encoding STAT4 (A), LAG-3 (B), and NKG-7 (C). (D) Gallein decreased MAP3K8 mRNA levels significantly relative to fluorescein-treated cells, but not to control cells. Gβ_1_ siRNA did not decrease MAP3K8 mRNA levels. (A-D) First set of graphs: box plots (top) and difference plots (bottom) show data from memory CD4^+^ T cells isolated from the peripheral blood of 20 healthy donors, stimulated with plate-bound anti-CD3 and soluble anti-CD28, and grown in conditions promoting TH2 differentiation in the absence or presence of gallein or fluorescein for three days. Second set of graphs: box plots (top) and difference plots (bottom) show data from memory CD4^+^ T cells isolated from the blood of 30 healthy donors and stimulated for three days with plate-bound anti-CD3 and soluble anti-CD28 in conditions promoting TH2 differentiation in the presence of Gβ_1_ siRNA or NT siRNA. mRNA levels were determined by qPCR. ^*^, *p* < 0.05; ^**^, *p* < 0.01; ^***^, *p* < 0.001; ^****^, *p* < 0.0001.

Gβγ inhibition significantly decreased the mean level of STAT4 mRNA in TCR-stimulated TH2 memory cells, consistent with the increased levels of mRNAs encoding TH2 cytokines. In these cells, gallein significantly decreased the mean level of STAT4 mRNA to 85% of the control value whereas fluorescein actually had the opposite effect, increasing the mean level of STAT4 mRNA to 107% (Fig. 6A). Similar to the effect of gallein, Gβ_1_ siRNA significantly decreased the mean level of STAT4 to 92% of the NT siRNA value (Fig. 6A).

In TH2 memory cells we also investigated whether Gβγ inhibition affected mRNA levels of the STAT4-regulated genes examined in TH1 cells, except for IL-18Rβ, as TH2 cells do not express the IL-18R [74], and OSM, which is expressed primarily in TH1 cells [82,83]. Release of soluble LAG-3-related peptides by activated CD4^+^ T cell clones was shown previously to correlate inversely with IL-4 production [77]. Consistent with this, gallein significantly decreased mean levels of LAG-3 mRNA in TCR-stimulated TH2 memory cells to 93% of the control values whereas fluorescein had the opposite effect, increasing mean levels of LAG-3 mRNA to 121% (Fig. 6B). Similar to the effect of gallein, Gβ_1_ siRNA significantly decreased the mean level of LAG-3 mRNA to 86% of the NT siRNA value (Fig. 6B). Gallein also significantly decreased the mean level of NKG7 mRNA in TCR-stimulated TH2 memory cells to 86% of the control value (Fig. 6C). Similarly, Gβ_1_ siRNA significantly decreased the mean level of NKG7 to 91% of the NT siRNA value (Fig. 6C). Although knockout of MAP3K8 promoted a TH2 cell response in ovalbumin-immunized mice [98], gallein did not significantly decrease the mean level of MAP3K8 mRNA in TCR-stimulated TH2 memory cells compared to untreated control cells, although there was a significant decrease to 91% of the level in fluorescein-treated cells (Fig. 6D). Gβ_1_ siRNA also did not have a significant effect on the mean level of MAP3K8 mRNA in TCR-stimulated TH2 memory cells (Fig. 6D).

## Discussion

**Figure 7:**
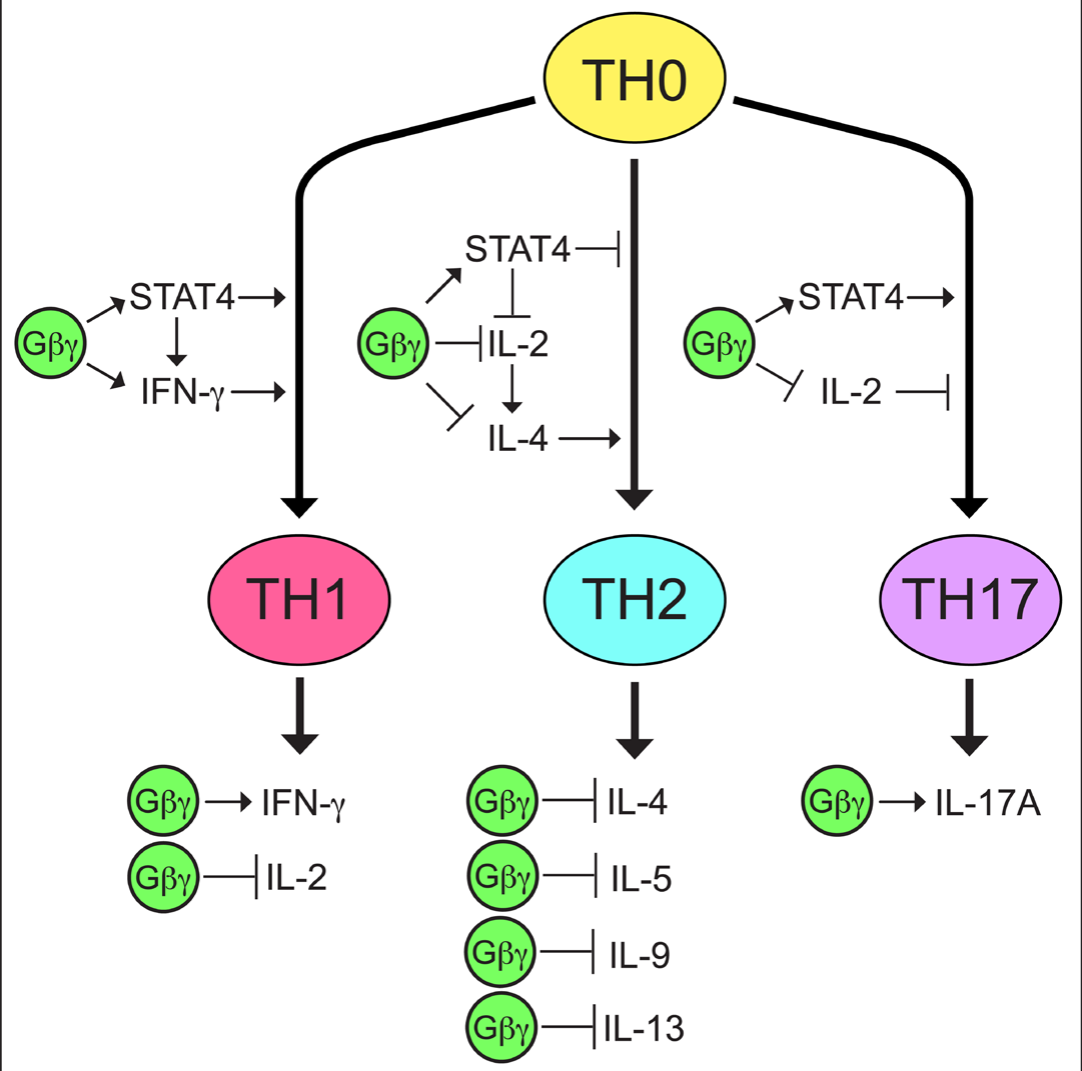
**Model of how Gβγ may contribute to proinflammatory cytokine production by CD4^+^ T helper cells.** There are multiple transcription factors and cytokines that influence the differentiation of CD4^+^ T helper cells, as well as cytokines that are produced by differentiated effector cells, but for simplicity, only STAT4 and the cytokines regulated by Gβγ are shown. Effects of Gβγ on IFN-γ, IL-4, IL-5, IL-9, IL-13, IL-17A, and STAT4 are based on the results of Gβγ inhibition on mRNA levels in the current study. The effect of Gβγ on IL-2 is based on previously reported results of Gβγ inhibition [1]. Previous reports described the roles of IFN-γ in TH1 differentiation [44], IL-2 in TH2 differentiation [31,102] and TH17 differentiation [12,13], IL-4 in TH2 differentiation [88,89], and STAT4 in the differentiation of TH1 [100], TH2 [97], and TH17 T helper cell subtypes [69,70].

The effects of Gβγ inhibition on cytokine mRNA levels in TCR-stimulated CD4^+^ T helper cells suggest that Gβγ signaling biases these cells in a proinflammatory direction by increasing levels of mRNA encoding IFN-γ, which is important for TH1 differentiation and function [44], decreasing levels of mRNA encoding IL-4, a TH2 cytokine that induces TH2 differentiation [88,89], as well as of IL-5, IL-9, and IL-13, which are also associated with TH2 responses [34,90,91,92], and increasing levels of mRNA encoding IL-17A, which exhibits proinflammatory activity [99] (Fig. 7). These effects on cytokine mRNA expression may be due in part to increased mRNA levels of STAT4 (Fig. 7), which is required for TH1 differentiation [100], inhibitory for TH2 differentiation [97], and can synergize with IL-23 and IL-18 to produce IL-17 [69].

Gβγ inhibition may lead to a shift away from production of proinflammatory TH1/TH17 cytokines towards TH2 cytokines by modulating pathways involving both STAT4, mRNA levels of which are regulated by Gβγ (Figs. 3, 6, and 7), and STAT5, activity of which is regulated by IL-2 [101]. The current study was initiated because inhibiting Gβγ signaling potentiated TCR-stimulated increases in IL-2 [1] (Fig. 7), which plays an essential role in TH2 differentiation, independent from effects on T cell proliferation, by stabilizing the accessibility of the *Il4* gene [31] and inducing IL-4 receptor α chain expression [102] via mechanisms involving STAT5. Regarding IL-17A, induction by IL-2 of STAT5 binding to the *IL17a* promoter was associated with a reduction in binding of STAT3 and the inhibition of associated active epigenetic marks [12]. Absence of IL-2 or disruption of its signaling by deletion of STAT5 resulted in enhanced TH17 differentiation [13]. However, IL-2 also promotes TH1 differentiation by inducing STAT5-dependent expression of the IL-12 receptor β2-chain (IL-12Rβ2) and the transcription factor T-bet [4]. Therefore, our observations that Gβγ inhibition both enhances TCR-stimulated IL-2 transcription [1] and decreases levels of IFN-γ mRNA in TCR-stimulated T cells (Fig. 1) might appear to be paradoxical. However, as STAT4 signals downstream of the IL-12 receptor, the decreased levels of STAT4 mRNA resulting from Gβγ inhibition could counteract the increased IL-12 signaling resulting from increased expression of IL-12Rβ2.

The decreased mRNA levels of STAT4 and STAT4-regulated genes resulting from Gβγ inhibition could lead to decreases in both IFN-γ and IL-17A by multiple mechanisms. STAT4 is required for IL-12-mediated induction of an IL-18R complex and IL-18Rβ [68], leading to synergistic induction of IFN-γ expression by IL-18 and IL-12 [65]. STAT4 also plays a role in the development of IL-23-primed IL-17-secreting cells, and is required for IL-17 production in response to IL-23 plus IL-18 [69]. Several of the products of STAT4-dependent genes that were decreased at the mRNA level in response to Gβγ inhibition play roles in the expression or function of these proinflammatory cytokines. For instance, in addition to its role in IFN-γ expression, IL-18Rβ is important for IL-17 expression, because IL-18 plays a role in activating/amplifying IL-17 production in polarized TH17 cells [75]. Moreover, deficiency of MAP3K8 is associated with impaired production of both IFN-γ and IL-17 [76]. Furthermore, simultaneous reduction of TCR-stimulated mRNA levels of IL-17A and OSM in response to Gβγ inhibition is clinically relevant because they synergize to induce cartilage breakdown [84,85] and cause skin inflammation [86].

There are many potential Gβγ effectors that could act alone or in combination to mediate a shift in TCR-stimulated CD4^+^ T cells from production of mRNAs encoding TH1/TH17 to TH2 cytokines. As gallein/M119 does not prevent interaction of Gβγ with N-type Ca^2+^ channels, inwardly rectifying K^+^ (GIRK) channels, ERK1/2, or adenylyl cyclase isoforms ACII, IV, and V1 [103], these effectors cannot account for the observed effects of Gβγ on cytokine mRNA levels. In contrast, Gβγ interaction with and activation of phosphatidylinositol-3-kinase-γ (PI3Kγ), P-Rex1, a Rac-specific guanine nucleotide exchange factor, PLCβ2/PLCβ3, and G protein-coupled receptor kinase 2 (GRK2) can be inhibited by gallein/M119 [103]. Moreover, gallein has been demonstrated to prevent Gβγ-mediated inhibition of Ca_V_1 Ca^2+^ channels [104], which in T cells are activated by the TCR by an unknown mechanism, rather than by T cell depolarization [105]. Additionally, Gβγ stimulates the Tek family kinase Itk [106]. Although the sensitivity of this interaction to gallein has not been tested, Itk is unlikely to mediate the cytokine shifts reported here because Itk plays a positive role in TH2 differentiation [107,108,109].

Of the known gallein-sensitive Gβγ-regulated effectors, PI3Kγ and P-Rex1 appear to be the most likely candidates for skewing T cells towards TH1/TH17 differentiation, based on some but not all previous studies. With respect to PI3Kγ, T cells from mice lacking PI3Kγ produced less IFN-γ in response to TCR stimulation [110]. Additionally, a selective PI3Kγ inhibitor blocked IL-17-producing TH17 cell differentiation in naive human CD4^+^ T cells [111]. However, another study reported that although PI3Kγ deficiency delayed the onset of experimental autoimmune encephalitis in mice, it was dispensable for TH1 and TH17 differentiation [112]. Additionally, pharmacological inhibition of PI3Kγ in murine lymph node T cells did not affect production of either IFN-γ or IL-5 [113]. Moreover, in human memory CD4^+^ T cells, PI3Kγ inhibitors reduced TCR-stimulated levels not only of IL-17A and IFN-γ, but also IL-4 and IL-13 [114]. In support of a role for P-Rex1 in mediating a shift from TH2 to TH1/TH17 cytokines, it is a nucleotide exchange factor that activates Rac1 [115] and Rac2 [116], and Rac2 plays a positive role in TH1 differentiation and IFN-γ gene expression, although the effect was more pronounced *in vitro* [117] than *in vivo* [118]. Moreover, CD4^+^ T cells from mice lacking Dock2, a Rac activator, exhibited excessive TH2 responses [119].

The remaining Gβγ-regulated effectors appear unlikely to mediate the cytokine shifts reported here for the following reasons. With respect to Ca_V_1 Ca^2+^ channels, which are important for Ca^2+^-mediated NFAT translocation to the nucleus [105,120] and which are inhibited by Gβγ [104], we demonstrated previously that Gβγ inhibition enhanced TCR-stimulated increases in intracellular Ca^2+^ and nuclear localization of NFAT1 [1]. However, it seems unlikely that these effects of Gβγ inhibition would produce a shift from TH1/TH17 cytokines to TH2 cytokines, as increased levels of intracellular Ca^2+^ appear to favor TH1 over TH2 differentiation [121,122] and NFAT1 deficiency enhances TH2 differentiation by prolonging IL-4 transcription [123,124,125]. PLCβ2 and PLCβ3 also appear unlikely to mediate the effects of Gβγ on cytokine mRNA levels because their activation by Gβγ leads to increased levels of intracellular Ca^2+^ in response to inositol trisphosphate [126,127]. Moreover, although PLC-γ plays an important role in T cell activation downstream of the TCR [128], PLCβ2/PLCβ3 are important for chemotaxis of lymphocytes but not for TCR-mediated T cell activation [129,130]. With respect to GRK2, upon GPCR-G protein activation, Gβγ can bind to the PH domains of GRK2/GRK3, causing translocation to the plasma membrane, and GPCR phosphorylation and desensitization [131]. Several previous reports suggest that blocking this interaction would be associated with increased rather than decreased levels of proinflammatory cytokines. For instance, decreased GRK2 expression was found in PBMCs from patients with RA [132] and MS [133,134]. Moreover, there is no simple scenario involving GRK2-GPCR regulation that can account for the similar effects of gallein and Gβ_1_ siRNA on levels of mRNA encoding IFN-γ, IL-17A, STAT4, or STAT4-regulated genes in TCR-stimulated TH1 cells. Blocking interaction between Gβγ and GRK2 with gallein might increase signaling of a GPCR that could decrease levels of these mRNAs, but Gβ_1_ siRNA would be predicted to decrease rather than increase signaling of this GPCR. However, in the case of mRNAs encoding IL-5, IL-9, and IL-13 in TCR-stimulated TH2 memory cells, where gallein, but not Gβ_1_ siRNA increased their levels, it is possible that blocking Gβγ-GRK2 interaction with gallein might increase signaling of a GPCR that had this effect. Further studies will be needed to identify the specific Gβγ-regulated effector(s) involved in shifting the cytokine mRNA profile in TCR-stimulated CD4^+^ T cells from proinflammatory TH1/TH17 towards TH2.

Additional studies will also be required to identify the GPCRs upstream of Gβγ that are involved in the regulation of the cytokine mRNAs studied here. Relevant to our observation of decreased levels of IL-17A mRNA upon Gβγ inhibition in TCR-stimulated cells differentiated under TH1 conditions, a population of cells that co-expressed CCR6 and CXCR3 produced both IL-17 and IFN-γ [52]. Additionally, CXCR4 and CXCR6 were found on TH17/TH1 clones [51]. As inhibiting Gβγ with gallein increased levels of mRNA encoding IL-4, IL-5, and IL-13 in memory but not naive TCR-stimulated TH2 cells, regulation of these cytokines may involve GPCR(s) expressed selectively in memory T cells, as there are differences in the GPCRs expressed in naïve versus memory CD4^+^ T cells [135]. For instance, deficiency of CCR2, which is expressed by both TH1 and TH2 cells [136] and is restricted to memory CD4^+^ T cells [137], led to an enhanced airway TH2 response in mice after allergen challenge [138]. Whereas disruption of the signaling of multiple GPCRs may be involved in the effects of Gβγ inhibition on the cytokine mRNAs reported here, it is also possible that the same GPCR is involved in regulating more than one cytokine. For instance, antagonizing EP2/EP4 receptors inhibited both TH1 differentiation and TH17 expansion [14,15]. Alternatively, the direct involvement of Gβγ in regulating cytokine expression does not necessarily implicate a GPCR [139]. For instance, in the absence of GPCR stimulation, AGS family proteins can activate Gβγ [140].

The decreased levels of mRNAs encoding IFN-γ and IL-17A resulting from Gβγ inhibition could have applications for autoimmune diseases. Whereas the effects of blocking Gβγ signaling on each of these cytokines were modest, they were comparable, as described in the Results section, to clinically relevant changes, and the combination of these effects might be useful because of the role that each of the cytokines plays in autoimmune diseases. Because elevations in both IFN-γ and IL-17 appear to play roles in the pathogenesis of RA [141], CD [142,143], psoriasis [144], MS [54,145], and HT [146], therapies focused on reducing both could be beneficial. “TH17/TH1” cells that produce both IFN-γ and IL-17 are particularly prominent at sites of inflammation such as the gut of patients with active CD [51] and in the brain tissue of MS patients [54]. Moreover, the results of one study suggested that IL-17 might play a crucial role primarily in the early phase of MS whereas IFN-γ might be involved both in the early phase and during relapses [145].

The increased levels of mRNAs encoding TH2 cytokines resulting from Gβγ inhibition could also be of therapeutic interest, as these cytokines can play protective roles against autoimmune diseases. For instance, IL-4 was decreased in human psoriasis patients [94] and IL-4 therapy induced TH2 responses and improved their clinical states [147]. IL-5 promoted induction of antigen-specific CD4^+^CD25^+^ T_Reg_ cells that suppress autoimmunity [148]. An anti-inflammatory role for IL-9 is supported by reports that IL-9 linked mast cells to T_Reg_-cell-mediated allograft tolerance [149,150]. IL-13 prevented autoimmune diabetes in NOD mice [151], protected against experimental autoimmune myocarditis by regulating macrophage differentiation [152], prolonged allograft survival [153], and attenuated acute kidney allograft injury [154]. In addition, IL-13 gene therapy in rheumatoid arthritis synovium reduced inflammatory cytokines and prostaglandin E_2_ [155]. Moreover, the effectiveness of current treatments for RA, such as anti-tumor necrosis factor and methotrexate therapy, may be due in part to inducing a TH2 shift [156,157], and inducing a TH2 shift has been proposed as a potential therapy for celiac disease [158], MS [159], kidney ischemia-reperfusion injury [160], and for prolongation of cardiac allograft viability [161].

## Conclusions

Inhibition of Gβγ signaling in TCR-stimulated CD4^+^ T Helper cells shifts the cytokine profile of these cells away from the proinflammatory TH1 and TH17 subtypes towards the TH2 subtype, both by decreasing mRNA levels of IFN-γ and IL-17A, and by increasing mRNA levels of TH2 cytokines. These changes correlate with and may result from increased levels of IL-2 and decreased levels of mRNAs encoding STAT4 and STAT4-regulated genes. Taken together, these results suggest that Gβγ may prove to be an attractive drug target for the many autoimmune diseases associated with increased levels of IFN-γ and IL-17A.

## Competing Interests

The authors declare that they have no competing interests.
